# Perinatal exposure to foodborne inorganic nanoparticles: A role in the susceptibility to food allergy?

**DOI:** 10.3389/falgy.2022.1067281

**Published:** 2022-12-05

**Authors:** Mohammad Issa, Gilles Rivière, Eric Houdeau, Karine Adel-Patient

**Affiliations:** ^1^ Département Médicaments et Technologies Pour la Santé (MTS), SPI/Laboratoire d’Immuno-Allergie Alimentaire, Université Paris-Saclay, CEA, INRAE, Gif-sur-Yvette, France; ^2^ French Agency for Food, Environmental and Occupational Health & Safety (ANSES, Agence Nationale De Sécurité Sanitaire De l’alimentation, De l’environnement et du Travail), Direction de l’Evaluation des Risques, Maisons-Alfort, France; ^3^ Toxalim UMR1331 (Research Centre in Food Toxicology), Toulouse University, INRAE, ENVT, INP-Purpan, UPS, Toulouse, France

**Keywords:** perinatal exposure, nanoparticles, food additives, intestinal homeostasis, food allergy

## Abstract

Food allergy (FA) is an inappropriate immune response against dietary antigens. Various environmental factors during perinatal life may alter the establishment of intestinal homeostasis, thereby predisposing individuals to the development of such immune-related diseases. Among these factors, recent studies have emphasized the chronic dietary exposure of the mother to foodborne inorganic nanoparticles (NP) such as nano-sized silicon dioxide (SiO_2_), titanium dioxide (TiO_2_) or silver (Ag). Indeed, there is growing evidence that these inorganic agents, used as food additives in various products, as processing aids during food manufacturing or in food contact materials, can cross the placental barrier and reach the developing fetus. Excretion in milk is also suggested, hence continuing to expose the neonate during a critical window of susceptibility. Due to their immunotoxical and biocidal properties, such exposure may disrupt the host-intestinal microbiota's beneficial exchanges and may interfere with intestinal barrier and gut-associated immune system development in fetuses then the neonates. The resulting dysregulated intestinal homeostasis in the infant may significantly impede the induction of oral tolerance, a crucial process of immune unresponsiveness to food antigens. The current review focuses on the possible impacts of perinatal exposure to foodborne NP during pregnancy and early life on the susceptibility to developing FA.

## Graphical abstract

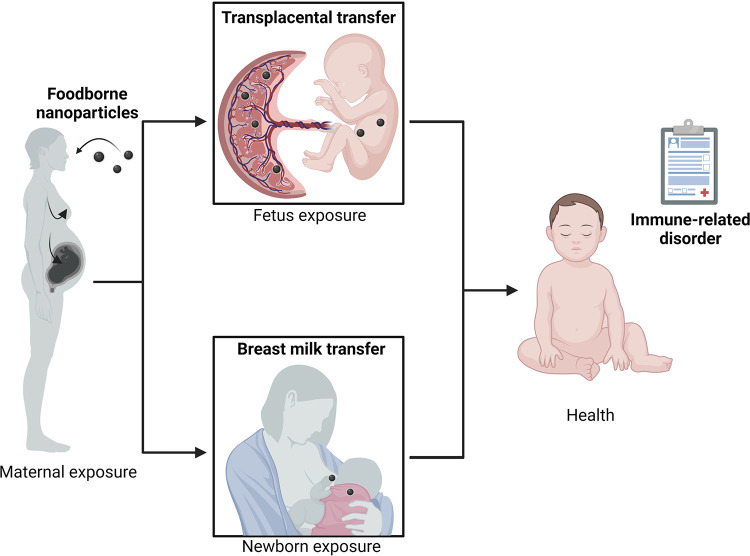

## Introduction

Nanotechnology is a fast-developing area in agricultural and food science. So far, nanotechnologies have brought significant improvements in the food sector by targeting agricultural production, manufacturing, food processing, packaging, safety, quality control, and food spoilage (1, 2). But such a rapid development and the now wide use of nanoparticle (NP)-based products in the human food chain raise issues for human health (3) and highlight the urgent need for a specific risk assessment. By focusing on immune-related hazards, numerous reports emphasized a large potential for immune-related consequences due to NP exposure through the diet (4). Based on these reports, the current review aims to assess the possible impacts of foodborne NP with regard to the risk of food allergy (FA) development.

## Food allergy: an immune system dysruption

### Food allergy vs. oral tolerance

FA is an adverse reaction that results from an inappropriate and excessive immune response against dietary proteins. This reproducible immune reaction results from an impaired induction of oral tolerance, i.e., a suppressive immune process at local and systemic levels that physiologically allows harmless dietary proteins to be tolerated by the immune system, thus avoiding chronic intestinal inflammation due to their regular consumption.

**Figure 1:**
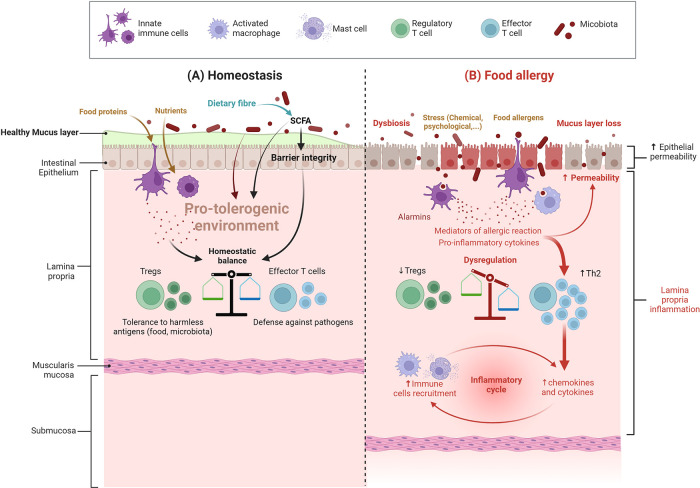
Intestinal barrier in a steady vs. food allergy state. (**A**) Under homeostatic conditions (cohesive intestinal barrier, diverse and active microbiota, exposure to food antigens), antigen-presenting cells promote the induction of food antigen-specific Treg cells. These cells induce tolerance to dietary antigens by a range of mechanisms, including inhibition of antigen-specific T helper type 2 (Th2) cell responses, suppression of pathogenic Th2 cell-like reprogramming of T cells and of mast cell activation, and the production of barrier-protective cytokines; SCFA: short-chain fatty acids (**B**) In food allergy, dysbiosis associated with an impaired gut barrier compromise the differentiation of naive T cells into Treg cells and instead leads to the differentiation of type 2 adaptive T helper cells (Th2) and inflammation – more details are provided in the text and in Figure 2 (Created with BioRender.com).

The establishment of oral tolerance is dependent on the controlled uptake of food proteins through the intestinal epithelial barrier and its delivery to local dendritic cells (DC) that matured in the intestinal pro-tolerogenic environment. The “pro-tolerogenic” antigen-loaded DC will migrate to draining mesenteric lymph nodes, where they will present the food protein-derived peptides to naive T cells and favour the induction of a subpopulation of T cells, namely regulatory T cells (Treg). These specific Treg will migrate back to the intestinal mucosa. Their further maturation and expansion will depend on food protein re-exposure. Some of these matured Treg will also migrate to other mucosal sites in the periphery (5, 6). Specific Treg then provide active tolerance to the harmless food proteins at all mucosal surfaces, either *via* cell contact or *via* their secretion of immunosuppressive factors that both prevent the induction of adaptive T helper (Th) lymphocytes in response to antigenic re-exposure (7). Efficient oral tolerance induction is then dependent on various factors, notably food antigen ingestion and the presence of a homeostatic pro-tolerogenic environment at the intestinal surface (Figure 1).

This pro-tolerogenic environment is dependent on many factors, such as intestinal barrier integrity and a well-matured and educated immune system, themselves depending on intrinsic (genetic) and extrinsic factors such as exposure to bioactive nutrients and antigens, as well as microbiota composition and function. On the other hand, environmental, pathophysiological, or specific exposure circumstances to other harmful conditions, such as various stressful life events, may disrupt oral tolerance induction, thereby paving the way to immune-related diseases such as FA.

**Figure 2:**
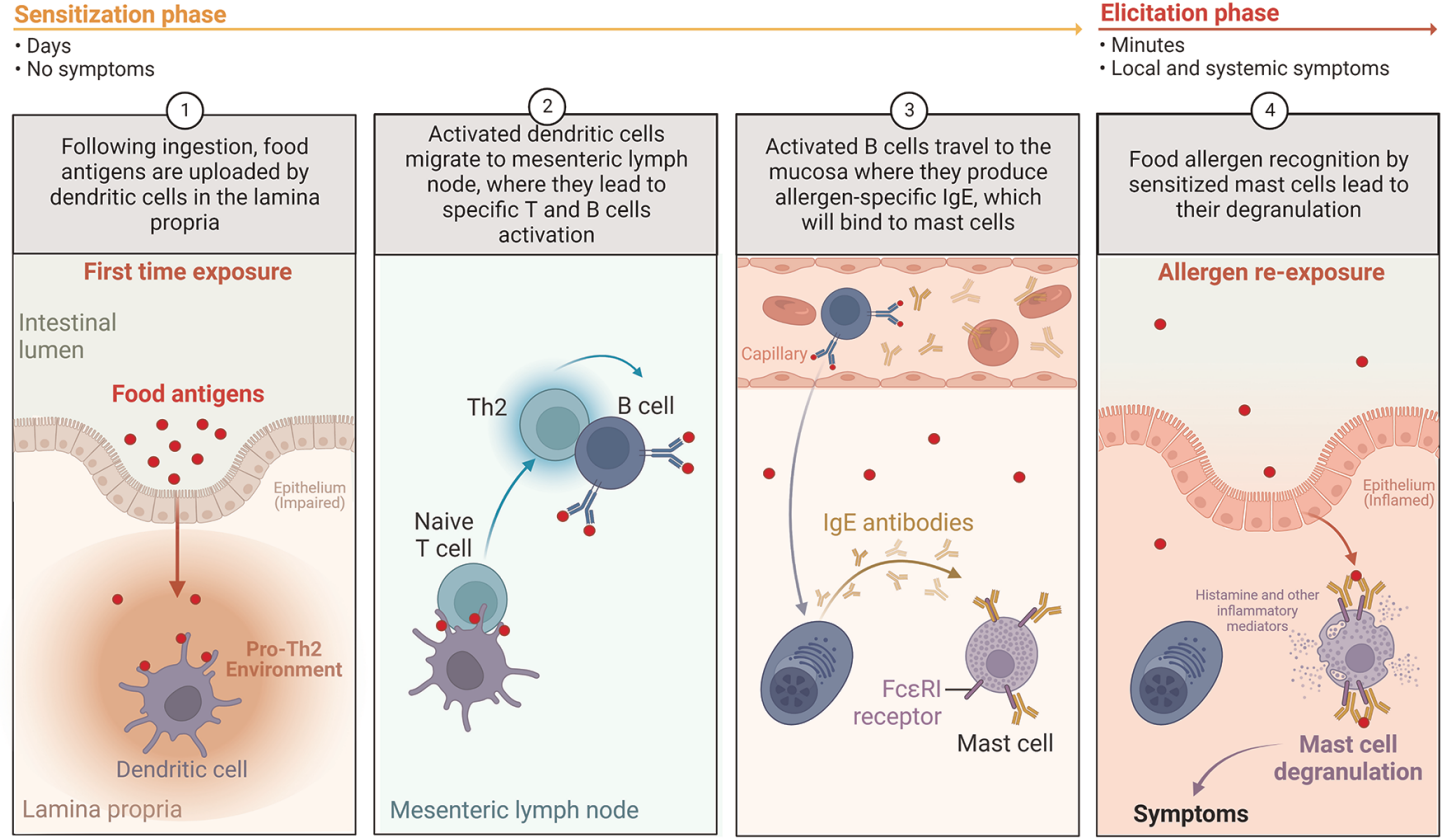
The development of IgE-mediated food allergy in humans. During the allergic “sensitization phase”, in the setting of an impaired barrier and a pro-Th2 environment: (1) following ingestion, food allergens are absorbed and uploaded by/delivered to dendritic cells in the lamina propria; (2) loaded dendritic cells migrate to mesenteric lymph nodes, where they prime naïve T cells in the presence of pro-Th2 cytokines. Th2 cells will activate antigen-specific B cells, leading to isotype switching to IgE; (3) Activated B cells will then differentiate into plasma cells and produce significant amounts of allergen-specific IgE (sIgE); (4) Secreted sIgE bind to high affinity Fc*ε*RI receptors on the surface of mucosal mast cells. When exposed to allergens again, allergen is recognized by sIgE bound to mast cells, leading to mast cell activation and release of preformed (histamine, tryptase, etc.) and *de novo*-synthesized (leukotrienes, prostaglandins, Th2 cytokines, etc.) pro-inflammatory mediators. This corresponds to the “elicitation phase” of the acute reaction, leading to Th2 local inflammation and clinical symptoms (Created with BioRender.com).

Indeed, FA relies on inappropriate activation of pro-inflammatory Th2-type responses against a food protein, called allergen. This response can occur at different mucosal surfaces (intestine, skin, respiratory) due to a pro-Th2 microenvironment associated with a barrier defect, thus leading to “allergic sensitization” instead of tolerance (7). Once sensitization has occurred, symptoms of FA will occur upon allergen re-exposure through ingestion of the offending food (Figure 2). FA involves type E immunoglobulin (IgE)-dependent and non-IgE mechanisms (8), which causes a variety of symptoms. Involvement of IgE and non-IgE mechanisms varies depending on age and country. The same food can cause one or both types of responses, as in the case of peanut (IgE) or cow's milk proteins (IgE or non-IgE) allergies (9, 10).

### Food allergy symptoms, prevalence and impact on quality of life

The symptoms of FA will vary depending on the underlying mechanisms (i.e., IgE or non-IgE), but also on what you are allergic to and how you come into contact with it [e.g., dose, matrix, but also (patho)physiological state]. In IgE-mediated FA, ingestion of the culprit food will rapidly lead to symptoms at various sites, affecting the gastro-intestinal tract (nausea, vomiting, diarrhoea), the skin (hives) and mucosa (tingling or itching in the mouth, swelling of the face, mouth, throat, or other areas of the body), or even the respiratory tract (wheezing, shortness of breath, asthma). Those reactions may occur alone or in combination. The more severe allergic reaction, called “anaphylactic shock”, can be life threatening. Non-IgE FA, such as food protein-induced enterocolitis syndrome (FPIES), food protein-induced allergic proctocolitis (FPIAP) or eosinophilic esophagitis (EoE), primarily affects the gastrointestinal tract and can be acute (FPIES) or chronic (FPIES, FPIAP, EoE) (11).

FA affects approximately 2%–5% of adults and 6%–8% of young children (12, 13). Egg, cow's milk, and peanut allergies are predominant in children (14). The estimated prevalence of FA is very variable and depends on many factors such as age, geographical location, eating habits (15) as well as definitions and criteria for diagnosis. Indeed, diagnosis can be based on either questionnaires, biological assays (specific IgE measurement), and/or deep allergological tests [skin tests, oral food challenge (OFC)] (16). Over the past two decades, the incidence of FA has steadily increased, notably in industrialized countries such as Australia (17–19), further underlining the role of environmental factors in this disease. Moreover, severity of FA reaction also increased: an increased number of hospitalizations due to food anaphylaxis is observed in several countries, as for example in England and Wales (+106% between 1998 and 2012), with a more marked increase among children under 14 years old (+137%) (20, 21). In France, according to data from the Allergo-Vigilance Network (22), the higher frequency of food anaphylaxis is observed for children under 10 years (34% of reported cases, especially in children under 3 years).

FA may have a dramatic impact on the quality of life of children and their families, particularly in terms of dietary habits, and psychological and socioeconomic aspects (23, 24). Atopy and the number of foods avoided are two factors that significantly affect general health perception, parent emotionality and family activities (25). The most significant factors affecting health-related quality of life in food allergic patients are perceived disease incidence, age of the patient, presence of peanut or soy allergy, country of origin, and having allergies to two or more foods (26). According to a Swedish study, older children (6–12 years) and those with severe FA have worse quality of life (24).

Finally, two small surveys collected data on a variety of direct FA care expenses, such as inpatient, outpatient, and prescription costs. They estimated a mean annual expense of $2,300 to $3,500 per patient per year, depending on age (27, 28). Household-level assessments of missed potential costs place the greatest economic impact of FA, with mean costs of $4,881 across many reports (29).

### Food allergy within the DOHAD concept

Since the incidence of food allergy peaks in childhood, one may hypothesize that environmental factors as soon as during early life may predispose to this immune-related pathology. FA thus has its place in the “Developmental Origins of Health and Disease” concept (DOHaD) (30) that emphasizes the role of prenatal and perinatal exposure to environmental factors in determining the development of human diseases. Indeed, the “first 1,000 days” of life, starting from conception, are known to represent a period of particular sensitivity to nutritional, metabolic and environmental (chemical or psychological stress) factors, the actions of which may lead to health concerns later in life. Within this window of susceptibility, the perinatal period has been defined by the World Health Organization (WHO) as the period between the twenty-eighth week of pregnancy (approximately 6 months) and the seventh day of life after birth. However, the term “perinatal” term generally covers a wider period (up to a year after giving birth) and refers to all events occurring during pregnancy, childbirth, and the neonatal period.

### Role of early diet in long-term allergic susceptibility

Different perinatal factors, alone or in combination, can then impair intestinal homeostasis (epithelial barrier, immune system, microbiota composition and function) and predispose to pathologies such as FA. These factors may act as early as *in utero*, as evidenced by defects in T cell function and epigenetic signatures already detected in cord blood samples and associated with FA at 12 months of age (31, 32). After birth, colonization by gut microbiota, feeding practices (breastfeeding vs. formulas, weaning and diversification practices), and exposure to various environmental chemicals – notably through diet, may further affect the establishment of intestinal homeostasis in the neonate. On this basis, the early life should be crucial for prevention vs. predisposition of the newborn to FA later in life.

Early-life dietary practices may have a long-term health impact. Exclusive breastfeeding is recommended for 4 to 6 months, covering all the nutritional needs of the developing neonate and providing efficient protection from infections and various immune/metabolic disorders such as asthma, diabetes or obesity (33). The composition of breast milk constantly changes over time to adapt to the nutritional needs of the infant. However, breast milk, and notably early breast milk (i.e., colostrum), can also influence the development and maturation of the barriers and immune system. This occurs *via* the transfer of bioactive components such as immunomodulatory cytokines, miRNAs, immunoglobulins and nutrients that act on the gut-associated lymphoid tissue (GALT), epithelial barrier and/or on the microbiota composition and function (34–38).

Interestingly, breast milk also contains dietary antigens ingested by the mother (39–41). Mouse model studies evidenced that milk-mediated transfer of an antigen to the neonate results in specific oral tolerance induction in the progeny (42), which may additionally depend on the immune status of the dam (43, 44). Indeed, the excretion of food antigens appears to be physiological and can play a role in educating the immune system toward specific tolerance. Those environmental antigens correspond to antigens to which the newborn will be exposed; as part of the mother's usual diet, they match the family's dietary habits.

Moreover, dietary antigen load is crucial for the maturation of the GALT, including the induction of Treg cells and oral tolerance. Actually, deprivation of food proteins in early life may alter the maturation of the immune system to the same extent as deprivation of the gut microbiota (45). Recent epidemiologic studies associate the use of infantile formulae based on partial hydrolysates at 2 months with the development of FA at 2 years of age (46). Using protein hydrolysates in early life also reduces their specific tolerating potency (43, 47). On the other side, the specific prevention of FA by the early introduction of food allergens has also been evidenced in various epidemiological and interventional studies (9, 48–50). In a randomized intervention study in children aged 4 to 11 months and at high allergy risk, early and regular ingestion of peanuts reduced by more than 70% the prevalence of food allergy to peanuts at 5-years old (LEAP study - Learning Early about Peanut Allergy (48, 51). Peanut ingestion by mothers while breast-feeding, combined with early peanut introduction in the first year of life, was associated with the lowest risk of peanut sensitization (52). However, Perkin and colleagues (53) found that the early introduction of six allergenic foods between 3 and 6 months of age, along with breastfeeding, did not prevent egg or peanut allergies.

Dietary practice in early life may then have a long-term effect on child immunity, notably FA development (54–56).

### The establishment of the gut microbiota: a key step in the maturation of the immune system

The gut microbiota gathers bacterial, fungus, archaeal, and virus communities that live in the gut in symbiosis with our organism. It is considered an organ due to its various functions, which participate in the (gut) homeostasis of the host. The gut microbiota exerts barrier activity against pathogenic microorganisms, has metabolic activity that provides essential nutrients and components to the host, and its permanent dialogue with the host's immune system participates in maintaining an effective response against pathogens while helping the induction of tolerance towards harmless antigens (57). Moreover, the presence of a diverse and rich intestinal microbiota is necessary for the proper development and maturation of the intestinal barrier and the GALT (58, 59). Numerous studies have shown that microbiota imbalance, namely dysbiosis, is associated with various pathologies (60). Studies have shown that gut microbiome functions are even more critical during early life (61–63). Indeed, early-life changes in the gut microbiome are associated with increased vulnerability to the development of FA, asthma, and autism later in life (64, 65).

The sequential establishment of the intestinal microbiota must then be tightly orchestrated. However, it can be impaired by various early life events such as prematurity, mode of delivery (vaginal vs. caesarean), diet (breastfeeding, weaning, and diversity practices), perinatal medication, and environmental microbiological richness. These factors may then induce early dysbiosis, such as reduced microbial diversity and alteration in the composition or function of certain bacterial communities, all of which could have long-term effects. For example, the mode of delivery will determine the initial profile of gut bacterial colonization (66), and caesarean delivery has been associated with a higher prevalence of FA in childhood (67). In adults who declared themselves allergic, intestinal dysbiosis was also detected but without knowing whether it was the cause or the consequence of the allergy (68).

Immune development and balance is then partially dependent on the symbiotic relationship of the immune system with the microbiota, and evidence now exists for a multidirectional interaction between the diet, the immune system, and the gut microbiota (69, 70). However, besides these interactions, immune system development is also exquisitely sensitive to nutritional factors and protein loads on its own.

In summary, the “first 1,000 days” of life represent a period of particular sensitivity to various environmental factors. However, to date, few studies have analyzed the impact of perinatal exposure to ultra-processed food as a source of foodborne inorganic NP on the immune system and microbiota development, and finally on FA development.

## Nanoparticles in the agrofood chain

**Figure 3:**
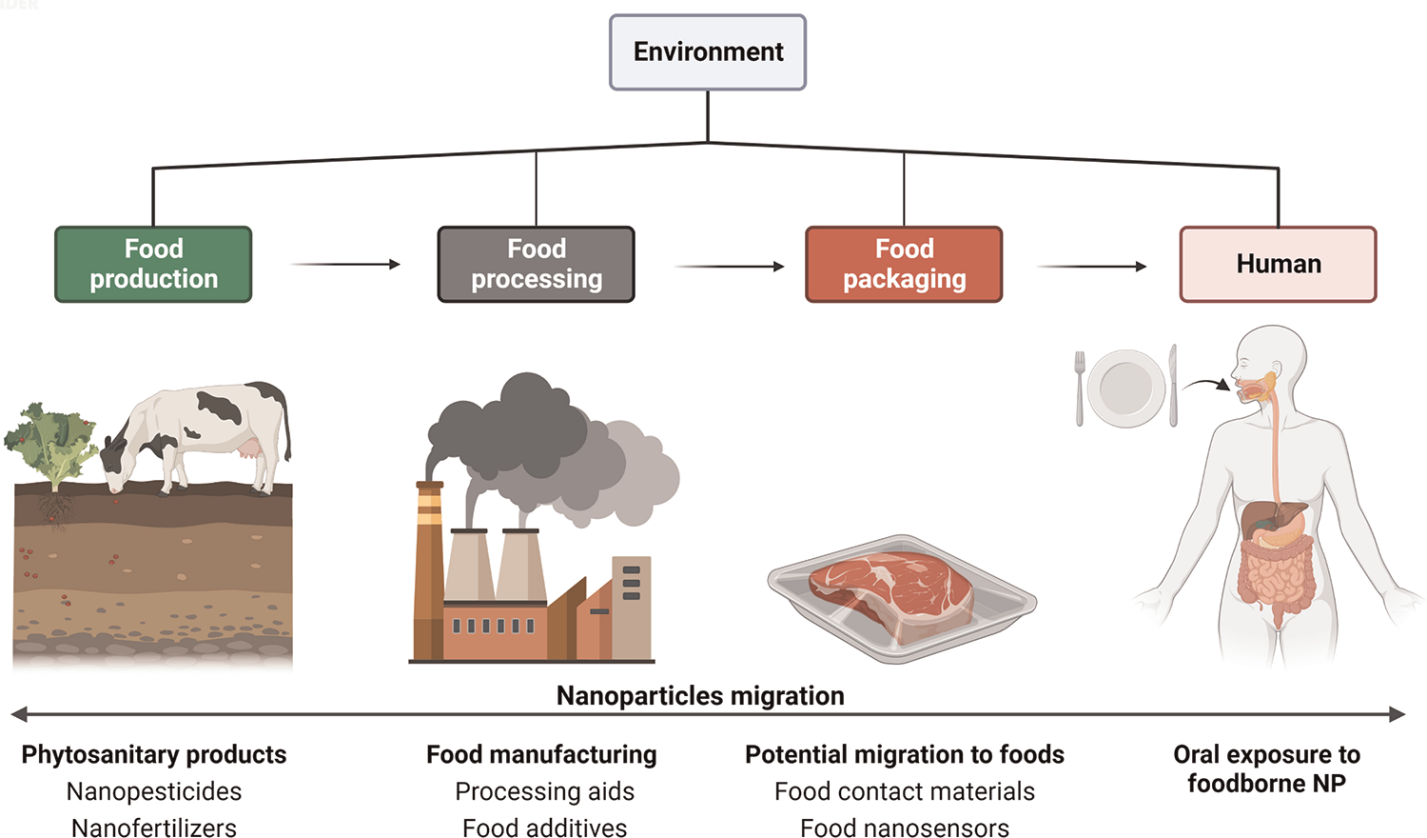
Applications of nanomaterials along the food chain. From left to right: Starting with the agricultural sector for food production, which uses nanoparticle (NP)-based formulations for innovative phytosanitary products (nano-sized pesticides, herbicides, fungicides, and fertilizers), second during food manufacturing with nanotechnologies applied from ingredients (food additives), processing aids (anti-caking agents, biocidal agents), until food packaging (new barrier properties for food contact materials, and nanosensors). Most of these applications can lead to chronic oral exposure of consumers through diet (Created with BioRender.com).

Since two decades, the use of nanomaterials [materials composed of particles with at least one dimension in the size range from 1 to 100 nm - (71)] spanned across various industries, including healthcare (e.g., nanomedicine, pharmaceutical products), cosmetics, and the agro-food chain. This is due to their specific properties linked to their size and physicochemical nature, which offer new opportunities for innovation in such sectors. For food, nanotechnology along the food supply chain has emerged in various forms. They are expected to have a beneficial influence on the enhancement of agricultural production (phytosanitary agents), as a pillar for sustainable agriculture. Other applications rapidly developed for food processing (processing aids and food additives) and packaging (food contact materials), as well as for food safety (e.g., nanosensors to detect foodborne pathogens), all favoring the shelf-life extension of food products while leading to chronic exposure of the consumers to NP (Figure 3). With concerns to FA predisposition in early life, assessing the impact of pre- and postnatal exposure to foodborne inorganic NP exhibiting immunomodulatory and biocidal activities appears clearly relevant.

### Phytosanitary products

Phytosanitary products are substances or mixtures of substances with chemical or biological properties used in agriculture, horticulture, or forestry, to protect cultivated plants and to treat their environment. The agrochemical industry is the first sector to show an increased interest in the use of nanotechnology, according to the French annual declaration of “substances in the nanoparticular state” (R-Nano register: https://www.r-nano.fr/). This concerns, for example, the development of new biocidal product formulations called nanopesticides, which correspond either to small particulates produced from an active ingredient of traditional pesticides (nano-emulsions, nano-suspensions) or to small structures like nano-spheres and micelles, which are used to encapsulate various active principles (72, 73). Furthermore, nutrients can be encapsulated or coated with nanomaterials for the controlled and gradual delivery of one or more nutrients, known as nanofertilizers (74). The increasing interest in the use of nanopesticides and nanofertilizers raises concerns about how environmental risk can be measured for regulatory purposes, including all the way up to humans (contamination of groundwater and of the food chain) (73, 75). Agrochemical nanoproducts might be considered particularly of concern for global health since they are a major diffuse and purposeful source of NP in the environment, including the food chain (76, 77).

### Processing aids

Processing aids are substances that are not consumed as food ingredients on their own, thus are not listed as food ingredients *per se* for the consumer, but which are deliberately used during the processing or transformation of raw materials, foodstuffs or their own ingredients in order to fulfil a given technological objective (78). Processing aids represent one of the applications of nanotechnology during food manufacturing, including anti-foaming and fining substances, biocidal and anti-caking agents. For example, silicon dioxide (SiO_2_) is added to powdered preparations (milk, salt, sugar, soups and spices) to keep dry ingredients free flowing and to prevent hardening during storage and packaging. Silicates are also used as a filter aid and anti-foaming agent in the beverage industry (79–82). Other examples are based on the antimicrobial activity of particles such as Ag-NP, one of the most widely used materials as a surface biocide on sieves for filtration and fining of a wide range of foods (liquid or solid). Corresponding Ag-NP have a spherical shape and a smooth surface. Under these conditions, Ag ions act as the biocidal substance to fight germs like bacteria, fungi, and yeasts, thus avoiding microbial contamination of food products during their processing (83–85). During food and beverage manufacturing, the use of processing aids may result in the unintentional, but technically unavoidable presence of NP and trace metal contaminants in the final processed foods (86).

### Food contact materials

Food contact materials (FCMs) are present throughout the food supply chain, from ingredient storage (tanks, silos) to food manufacturing (worktops, conveyor belts, machines), as well as in the packaging, jars or boxes that contain the final processed foods and beverages (87, 88). Coating the walls of freezers and refrigerators with antibacterial NP such as metals and metal oxides (nano-Ag, ZnO, and TiO_2_, the latter being biocidal by photocatalysis) also falls into the category of FCMs. The technological gain here is to apply a surface biocide capable of preventing the development of bacteria, fungi and viruses in these food storage enclosures (87, 89, 90). Adding Ag-NP directly to final food packaging also gives it biocidal properties to protect packaged foodstuffs from potential bacterial contamination (91). We also find FCMs in “intelligent” labelling, i.e., with nanosensors in direct contact with food that aim to inform consumers on the state of preservation of foodstuffs by detecting microbial contamination, rotting or aromas representative of the state of maturity of the packaged product. These technologies notably use NP that change of color by oxidation, as in the example of inks detecting oxygen and containing TiO_2_-NP sensitive to light. Nanotechnology makes it possible to miniaturize these processes, which can then be incorporated into conventional labels or placed on the packaging itself.

Other applications of NPs such as FCM arise from the desire to ban plastics of petrochemical origin, which paved the way for the development of biodegradable materials from renewable sources but whose current weakness is the loss of barrier properties to protect food from degradation and contamination. Nanoparticles incorporated into these new materials, i.e., as nano-composites, offer multiple improvements, allowing the increase and best use of the original functions of packaging, i.e., protecting/preserving food and improving its preservation (91, 92). Nano-composite materials allow a gain in mechanical resistance (for light and rigid bottles), and in sealing (UV, water, gas). For example, nano-clays (e.g., montmorillonite) are incorporated into the thickness of the support material to limit the passage of oxygen to the food (92). Nanosized metals and metal oxides are also mixed with thermoplastic polymers and biopolymers to enhance their barrier properties, for example by reducing their permeability to oxygen, humidity, and CO_2_ (92, 93).

Whatever the field of application, a wide range of mechanical, physical and biochemical properties is made possible with nanotechnologies and is a core of development for most of the manufacturers concerned. The question of the health risk of NP migration into food is thus raised for the scientific community, and the health authorities, but is still poorly documented.

### Food additives

Food additives are organic or inorganic substances, solid or liquid, intentionally added to foodstuff as ingredients for various technological functions and properties, such as guaranteeing food safety (preservatives, antioxidants), improving the palatability and appearance (colorants, sweeteners, flavor enhancers), providing a certain texture (thickeners, gelling agents), or ensuring product stability (emulsifiers, anti-caking agents, stabilizers). Their presence in meals is indicated in UE in the ingredient list by either their code (E followed by 3 or 4 numbers) or their name (91, 94). In Europe, regulations EC/1331/2008 and EC/1333/2008 frame and standardize the examination and licensing of food additives at the EU level, and their conditions of use are reassessed as needed by the European Food Safety Authority (EFSA).

Among the ∼350 food additives authorized in the EU, about 10% are inorganic substances composed of NP. Nanoparticular structure is confirmed by transmission electron microscopy (TEM) to ensure the presence of nano-sized particles among their components, or suspected on the basis of their mode of production and use in the absence of specific data in the literature or provided by manufacturers (94). They are found in texturing agents (anti-caking and anti-foaming) that are directly added as an ingredient (in contrast to processing aids) to improve the fluidity of food powders such as sugar, salts, chocolate powder, freeze-dried soups, or spices. The most representative are silicon dioxide (SiO_2_, listed as E551) and aluminum silicate (E559). Nanoparticles are also found in food colorings, the best known being the white pigment and opacifying agent titanium dioxide (TiO_2_, E171), but also iron oxides (FeO, E172) with a black, red or yellow color depending on the state of oxidation. Of note, TiO_2_ is employed in a wide range of other industrial applications due to its coloring and opacifying properties (e.g., cosmetics, personal care products, pharmaceuticals and paints), as well as for biocidal activities due to its photocatalytic activity with TiO_2_ incorporated into various building materials (95, 96). Thus, human exposure to TiO_2_-NP occurs through inhalation and dermal contact in addition to the oral route. However, daily oral exposure to E171 is considered as the main source of body contamination to TiO_2_-NP in the general population (97). Nano-sized particles may also be present in edible silver (E174) and gold (E175) additives used for cake and confectionery decoration, as well as in spirit drinks. Another common ingredient is calcium carbonate (E170), an acidity regulator also used for its anti-caking properties (98). This list also includes phosphate-based food additives such as tricalcium phosphate (E341), a firming, leavening, and anti-caking agent also used as a thickener, humectant, acidity regulator, emulsifying salt, sequestering agent, and stabilizer (99), and calcium silicate (E552) with an anti-caking function (100). Among other inorganic substances suspected to contain NP, magnesium phosphates, ferric ammonium citrate, sodium, potassium, and calcium salts of fatty acids have been listed (94).

According to the food composition databases OQALI (French Observatory of Food) and GNPD (Global New Products Database), over 900 food products include at least one additive or component that belongs under the category “substances for which the existence of produced nanomaterials has been proven.” The most concerned food sub-sectors are infant formula (25.6%), confectionery (15.6%), breakfast cereals (14.8%), cereal bars (12.9%), frozen pastries and desserts (10.9%). The current review will focus on three NP-containing food additives (E171, E551 and E174) among the most often used in these food categories, as presented in Table 1, and for which significant effects on intestinal homeostasis establishment can be suspected based on available literature.

**Table 1:** Summary of common food-related usages of E171, E174 and E551, and of total estimated exposure in humans in a maximum level exposure scenario per group of age. I: Infants (12 weeks–11 months), C: Children (3–9 years), A: Adults (18–64 years) in mean (minimum-maximum) across the dietary surveys in mg/kg or µg/Kg body weight (bw)/day, according to EFSA risk assessment in EU.

	E171 Titanium dioxide	E174 Nano-silver	E551 Silicon dioxide
Food-related use	Whitening and opacifying	Food coloring	Anti-caking and texturing
Nanoparticles	TiO_2_	Ag	SiO_2_
Maximum level exposure assessment scenario (min-max by group of age)	I: 0.06–3.6 mg/kg bw/day C: 1.9–11.5 mg/kg bw/day A: 0.7–6.7 mg/kg bw/day	I: 0.01–0.77 µg/kg bw/day C: 0.22–2.6 µg/kg bw/day A: 0.03–0.65 µg/kg bw/day	I: 18.5–74.2 mg/kg bw/day C: 10.2–31.2 mg/kg bw/day A: 4.9–13.2 mg/kg bw/day
Main food categories contributing to exposure	- Infant Formula - Bakery wares - Soups - Broths - Sauces	- Confectionary - Decorations - Coating and fillings - Spirit drinks - Breath fresheners	- Dried powdered foods - Sugar and syrups - Fine bakery wares - Ripened cheese
EU safety assessment	(101)	(102)	(103)

## Does perinatal exposure to foodborne NP may increase the risk of food allergy?

As already noticed, the perinatal period is a critical window of vulnerability during which exposure to potentially harmful chemicals, and potentially NP, may increase the susceptibility to immune-related disorders, among other effects on the progeny.

### Placental and breast milk transfer of nanoparticles

The placenta is a temporary and multifunctional organ that acts as a barrier between the mother and the fetus, while it also regulates the exchange of nutrients and waste products. Whether foodborne NP may cross the placental barrier and may pose risks to the growing fetus is still being investigated. In humans, studies have been conducted *in vitro* on trophoblastic cells and *ex vivo* using isolated and perfused placenta (104–106). They demonstrated that the size is an important contributing factor for particle transfer to the fetal compartment. Indeed, *ex vivo*, only polystyrene beads up to 240 nm crossed the human syncytiotrophoblast that separates the fetal circulation from the maternal blood (106). Although the transfer rate appeared low using *ex vivo* placental perfusion, this suggested the capacity for the nano-sized fraction of food additives coming from the mother's diet to reach the fetus throughout the whole pregnancy period. For example, using models of NP of various sizes, transplacental passage of TiO_2_-NP has been reported in rats and mice (107–112). Moreover, using TEM combined with Ti element dosage, a recent study in humans clearly evidenced TiO_2_ particles in the human placenta and meconium, i.e., the first stools of the infant, depicting fetal exposure (105). Of interest, a non-negligible fraction of NP is probably of foodborne origin, as demonstrated using isolated human placenta perfused with a E171 suspension, concluding on a materno-fetal transfer of NP matter from the food-grade form of TiO_2_ (105). Furthermore, oral administration of TiO_2_-NP to lactating mice increases the NP concentration in milk, as observed after airway exposure (113). Altogether, this suggests an additional transfer of NP of foodborne origin to the newborn during breastfeeding, along with possible other environmental sources of NP inhaled by the mother and recovered in milk.

The transplacental passage of SiO_2_-NP has been investigated using the BeWo b30 choriocarcinoma cell line (placental trophoblast monolayer) (114). The authors clearly showed that SiO_2_-NP of 25 and 50 nm are able to cross the placental barrier, a passage confirmed *ex vivo* in the same study using perfused human placenta. Consistently, SiO_2_-NP administered to mice at various gestational periods reached the placenta and fetus, their biodistribution being driven by NP size and gestational stage (115). Both SiO_2_- and TiO_2_-NP, with diameters of 70 nm and 35 nm, respectively, can cause pregnancy complications when injected intravenously into pregnant mice. Indeed, both of these NP were recovered in the placenta, fetal liver, and fetal brain, along with smaller *uteri* and fetuses in the treated mice (111). To date, it has not been investigated whether SiO_2_-NP distributed in mother's blood also translocate to breast milk (103).

For assessment of Ag-NP transfer, both animal and *in vitro* studies concluded on a transplacental passage (116–121), an observation also confirmed using the perfusion model of the human placenta (122). During lactation, Ag-NP is excreted in milk after intravenous or oral administration to lactating mice, with Ag-NP recovered in the brain of breast-fed pups (123). Previous quantitative assessments of the NP transfer during breastfeeding in rats reported that the total accumulation of Ag-NP in the milk exceeded 1.9% of the administered dose over a 48 h period (14–16th day of lactation), and that not less than 25% of this amount was absorbed into the gut of rat pups (124).

Other studies show that the placenta is unable to completely prevent the passage of other engineered NP of possible dietary origin, such as gold, iron oxide, zinc oxide, and aluminium oxide (125, 126), with NP exposure continuing during breastfeeding (113). All these findings point to interactions as early as *in utero* between foodborne NP from the mother's diet and the fetal then the newborn's developing organs, including the gut barrier and its associated immune system.

### Impacts on gut microbiota

Colonized at birth, the human gastrointestinal tract harbors more than 7,000 strains and several hundred species, a majority of the bacteria belong to the phyla Firmicutes and Bacteroidetes, representing approximately 90% of the microbial population. Other species belong to the phyla Proteobacteria, Verrucomicrobia, Actinobacteria, Fusobacteria, and Cyanobacteria (127). A consensus exists that such a complex bacterial community has essential roles in digestion and fermentation of indigestible polysaccharides, production of vitamins, while they are also crucial for the development and maintenance of the gut barrier function (128–130). Inorganic NP exhibiting biocidal properties, mainly metals and metal oxides, may potentially interfere with the establishment of gut microbiota in offspring. This could occur by interacting with the metabolic activity and bacterial composition of the mother's microbiota, which is transmitted to the baby after vaginal delivery (131), a primocolonization that contributes to epithelial maturation of the offspring intestine (132) and the concomitant development of immune (GALT) functions (130, 133). During breastfeeding, translocation of biocidal NP to maternal milk could worsen the situation by progressively interfering with the establishment of homeostasis of this microbial ecosystem in terms of population, quality and activity (also called eubiosis). While chemical substances present in food, such as emulsifiers (134) or sweeteners (135), can cause intestinal dysbiosis, the question of the possible effect of foodborne NP on the gut microbiota has been posed only very recently (4).

To decipher NP impact, the main challenge focuses on food-grade TiO_2_, SiO_2_ and Ag which are suspected of altering the composition and/or activity of intestinal microbiota due to biocidal properties and accumulation in the gut lumen (4, 136). Indeed, the poor absorption rate of SiO_2_ (E551) in the human GI tract (103), along with toxicokinetic studies on TiO_2_ particulate matter, showed that at least 99% of these food additives are not absorbed and accumulate in the gut lumen (137–139). This highlighted a long-term interaction of foodborne NP with gut bacteria, and the potential for alterations in the growth profiles of bacteria, as observed *in vitro* for E171 (140). Size and shape of NP are determining factors in the potential for interaction with the gut microbiota because they affect the surface area/volume ratio and ionization potential (141) (142, 143). Changes in Firmicutes/Bacteroidetes (F/B) ratio, a solid indicator for composition changes in the gut, have been repeatedly observed in the faecal microbiota of rats and mice orally exposed to Ag-NP for varying durations and doses (142–145). In addition, a disturbance in the metabolic activity of the gut microbiota was reported in adult mice exposed to E171, resulting in the deregulation of host signalling pathways (146). Finally, one study in mice exposed to SiO_2_-NP for one week at a human-relevant dose reported increased proportions of Firmicutes and Proteobacteria, and decreased proportions of Bacteroidetes (142).

Overall, the literature suggests an impact of Ag-, TiO_2_-, and SiO_2_-NP on microbiota composition and global function, with a dysbiosis characterized by a change in the F/B ratio, a decrease in *Lactobacillus*, and an increase in Proteobacteria (4). It is worth noting that a shift in the F/B ratio has also been observed in dysbiosis associated with FA (147). On this basis, studies suggest that foodborne NP from common food additives have the potency to disrupt homeostasis in the intestinal microenvironment, impairing the establishment of the microbiota and then the maturation of the epithelial barrier and immune function during perinatal life.

### Impacts on gut barrier integrity

As stated above, although oral bioavailability studies in rodents and humans clearly established very limited absorption of foodborne inorganic particles by the gut, e.g., less than 1% of the initial dose for TiO_2_ and SiO_2_ (4) this represents billions of NP due to chronic oral exposure through the diet. Translocation and then accumulation of NP downstream in the gut mucosa may alter intestinal permeability, thus participating in disrupting intestinal homeostasis, which can circumvent the control passage of food antigen needed for oral tolerance induction. Evidence was first obtained for TiO_2_-NP models and, more recently, the food form E171 of TiO_2_. Indeed, *in vitro* studies using Caco-2 cells as an enterocyte model showed that TiO_2_-NP disturb tight junctions (TJs) that control paracellular spaces, hence increasing epithelial permeability along the intestine. This effect was observable as soon as 4 h post-exposure, with a broad impact on barrier integrity at 24 h (148). Several *in vivo* studies also demonstrated that nano-sized TiO_2_ particles exert detrimental effects on the intestinal epithelium layer. As example, increased epithelial permeability leading to impaired barrier function as well as immune damage have been reported after oral exposure of juvenile mice for 28 days to foodborne TiO_2_-NP at doses close to human dietary levels (149). Whether such changes may impact allergen exposure has been recently addressed *in vitro* using a co-culture model composed of Caco-2 cell monolayer exposed to allergy sera-primed mast cells. Authors showed that particle treatment with TiO_2_-, SiO_2_- or Ag-NP increased allergen delivery across epithelial layer through remodelling of TJs complex, and triggered allergic responses in pre-sensitized mast cells when exposed to milk allergens (150).

### Impacts on GALT functions

Although the translocation mechanisms of inorganic NP (including from food additives) in the intestine are still being discussed (151, 152), once absorbed, NP can directly interact with immune cells of the GALT which are essential in the establishment of tolerance to food antigens.

In the small intestine, the particulate characteristics of NP may enhance their upload by the Peyer's patches (PP) through Microfold (M) cells present in the dome of PP, a passage involved in the sensitization to food antigens (153). Indeed, M cells are specialized in capturing luminal antigens to present them to downstream immune cells (lymphocytes, macrophages and dendritic cells), thus triggering an appropriate immune response (tolerogenic or defensive) depending on the nature of the captured antigen. In rats and mice, accumulation of the Ti element in PP has been observed following ingestion of TiO_2_ nanomodels or the food additive E171 (154, 155). Similar accumulation sites have been reported in humans for Ti, together with silicate and aluminium particulate matter of foodborne origin (156, 157). In the ileum and outside PP sites, NP may also translocate through epithelial cells (enterocytes) to directly reach the mucosa, as observed with E171 and TiO_2_-NP models (151, 152). Oral administration of Ag-NP in rats also results in an accumulation of NP in the ileal tissues, more specifically in the lysosomes of the *lamina propria* (LP), in macrophages, and in the submucosa (158). Finally, intestinal absorption of SiO_2_- and TiO_2_-NP also occurred in the distal colon (152, 154), a region where GALT is crucial for the tolerance towards gut microbiota and host defences (4).

Despite several *in vitro* and *in vivo* studies emphasizing nano-TiO_2_, SiO_2_ and Ag with immunotoxic effects on blood, lung, or bone marrow-derived immune lines or cells (4), studies specifically focused on GALT are rare. In rats orally exposed to a human- relevant dose of E171, an accumulation of dendritic cells (antigen-presenting cells) in the PP was reported after 1 week of exposure (154). In the same study, a decreased frequency of regulatory T cells (Treg) involved in oral tolerance occurred in PP after 1 week of exposure to E171, an effect still observed following 100 days of treatment. In parallel, a Th1-mediated inflammatory response was observed in the small bowel of adult mice exposed for 10 days to TiO_2_-NP (159), whereas chronic exposure for 100 days to the food-grade form (E171) led to low-grade inflammation and immunosuppression in the colon (154, 160). Further studies have confirmed the down-expression of genes involved in the innate and adaptive immune responses in the distal colon of mice treated for 21 days with E171 (161).

Immunotoxicity of Ag-NP was also assessed in rats or mice (141, 143, 162), using various methods of administration (gastric gavage, addition to drinking water, or to food), although the doses often exceed the estimated daily intake in humans (0.001 mg/kg bw/day). As an example, a 28 days oral exposure in rats (9–36 mg/kg bw weight/day) evidenced decreased intestinal gene expression involved in T-cell regulation (*FOXP3*, *GPR43*, *IL-10* and *TGF-β*) (141). In a similar experiment at lower doses (0.25–1 mg/kg bw/day), an increased frequency of B-cells was reported, together with a dose-dependent increase in inflammatory cytokines levels in blood (163). Interestingly, in this later study by Park and collaborators, increased IgE antibodies concentrations in blood were also noticed. Finally, mice exposed to SiO_2_-NP showed a blockade of tolerogenic mechanisms towards a food antigen model (ovalbumin, OVA) (164), and co-administration of SiO_2_-NP with OVA can generate OVA-specific Th2-type immunological responses in female BALB/c mice after intranasal administration (165), thus suggesting a Th2 adjuvant capacity of these NP in the airway.

Altogether, this suggests foodborne NP may alter immune homeostasis and mechanisms of tolerance induction and favor sensitization to food proteins at mucosal sites. But foodborne NP may also impact the elicitation phase of allergy. Indeed, using the rat basophilic leukemia RBL-2H3 (a cell line commonly used as histamine-releasing cell line in inflammation and allergy), and primary mouse bone marrow-derived mast cells (BMMCs), exposure to TiO_2_-NP increased the IgE-dependent mast cell degranulation (166). Chen and colleagues (167) also observed that a mixture of anatase and rutile TiO_2_-NP (i.e., the two crystal forms authorized as food-grade TiO_2_) can directly stimulate histamine release from non-activated RBL-2H3 cells. Finally, consistent with SiO_2_ studies, the impact on mast cell degranulation was assessed using Ag-NP, showing they were able to elicit bone marrow-derived mast cell activation (168).

## Concluding remarks

**Figure 4:**
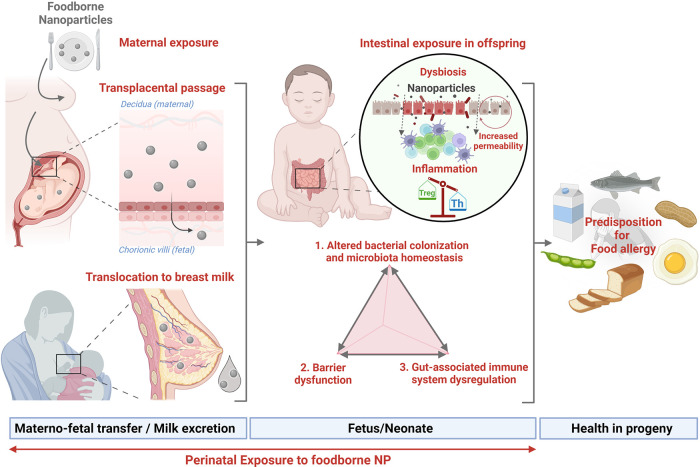
Perinatal exposure to nanoparticles and susceptibility to food allergy (created with bioRender.com).

The perinatal period is considered a critical window of increased susceptibility to nutritional, metabolic, and environmental factors that might influence the individual's health. Among the environmental factors, foodborne inorganic NP present in ultra-processed food exhibit a large spectrum of intrinsic (physico-chemical) properties able to imbalance essential components of intestinal homeostasis, including microbiota composition and function, gut barrier integrity, and the local immune system (GALT), which may predispose the progeny to chronic diseases related to immune dysregulation, such as FA. Metal oxides and silicate NP such as TiO_2_, SiO_2_, and Ag may cross the placental barrier and be excreted in breast milk, as illustrated in Figure 4. Their anti-microbial effects could impair microbiota set-up in early life and the concomitant maturation of immune and epithelial barrier functions starting at birth, whose development continues throughout the neonatal period. The consequences of early exposure to NP during the “first 1,000 days” of life require further studies to decipher whether perinatal NP exposure could predispose to the development of FA among other immune-related disorders. Additional studies are thus urgently needed to quantify and further characterize the human fetal and neonatal exposure to NP, and to determine the potential hazard for fetal/neonate development and their long-term health effects.
